# Regulatory T Cells Contribute to the Inhibition of Radiation-Induced Acute Lung Inflammation via Bee Venom Phospholipase A_2_ in Mice

**DOI:** 10.3390/toxins8050131

**Published:** 2016-04-30

**Authors:** Dasom Shin, Gihyun Lee, Sung-Hwa Sohn, Soojin Park, Kyung-Hwa Jung, Ji Min Lee, Jieun Yang, Jaeho Cho, Hyunsu Bae

**Affiliations:** 1 Department of Physiology, College of Korean Medicine, Kyung Hee University, Seoul 130-701, South Korea; ssd060@naver.com (D.S.); glee@khu.ac.kr (G.L.); ih_sj@naver.com (S.P.); jhkh242@naver.com (K.-H.J.); yje2020@hanmail.net (J.Y.); 2 Department of Radiation Oncology, Yonsei University College of Medicine, Seoul 120-752, South Korea; 19980769@hanmail.net (S.-H.S.); carol114@yuhs.ac (J.M.L.)

**Keywords:** bee venom, phospholipase A_2_, inflammation, radiotherapy, regulatory T cells

## Abstract

Bee venom has long been used to treat various inflammatory diseases, such as rheumatoid arthritis and multiple sclerosis. Previously, we reported that bee venom phospholipase A_2_ (bvPLA_2_) has an anti-inflammatory effect through the induction of regulatory T cells. Radiotherapy is a common anti-cancer method, but often causes adverse effects, such as inflammation. This study was conducted to evaluate the protective effects of bvPLA_2_ in radiation-induced acute lung inflammation. Mice were focally irradiated with 75 Gy of X-rays in the lung and administered bvPLA_2_ six times after radiation. To evaluate the level of inflammation, the number of immune cells, mRNA level of inflammatory cytokine, and histological changes in the lung were measured. BvPLA_2_ treatment reduced the accumulation of immune cells, such as macrophages, neutrophils, lymphocytes, and eosinophils. In addition, bvPLA_2_ treatment decreased inflammasome-, chemokine-, cytokine- and fibrosis-related genes’ mRNA expression. The histological results also demonstrated the attenuating effect of bvPLA_2_ on radiation-induced lung inflammation. Furthermore, regulatory T cell depletion abolished the therapeutic effects of bvPLA_2_ in radiation-induced pneumonitis, implicating the anti-inflammatory effects of bvPLA_2_ are dependent upon regulatory T cells. These results support the therapeutic potential of bvPLA_2_ in radiation pneumonitis and fibrosis treatments.

## 1. Introduction

Bee venom (apitoxin) has long been used in alternative medicine to treat various diseases, such as rheumatoid arthritis, asthma, cancer, and neurodegenerative diseases, and possesses strong immune modulatory effects [1,2,3,4,5,6]. The therapeutic effect of bee venom is associated with its anti-inflammatory, anti-cancer, and anti-nociceptive activity. Bee venom consists of phospholipase A_2_ (bvPLA_2_), melittin, adolapin, apamin, and mast cell degranulating peptide [7]. BvPLA_2_ has been considered as a major allergenic compound of bee venom; however, new experimental data has shown the protective immune responses of bvPLA_2_ against a wide range of diseases, including inflammatory disorders [8].

Radiotherapy (Irradiation; IR) is a treatment involving the use of high-energy radiation, commonly used to treat cancer. It can also be used prior to surgery to shrink a tumor so that it is easier to remove, or post-surgery to destroy small amounts of tumor that may be left. Stereotactic body radiotherapy (SBRT) delivers a higher dose of radiation with improved accuracy, compared to conventional radiotherapy, and many SBRT studies in lung cancer have reported excellent results [9,10]. Nevertheless, the adverse effects of radiation on normal tissues that surround the tumor often preclude the application of curative radiation doses. These radiation-induced side effects, which include radiation pneumonitis and late pulmonary fibrosis, are common. In a previous study, it was shown that radiation pneumonitis and late pulmonary fibrosis may involve inflammasomes and an innate immune response to tissue inflammation and injury [11,12,13]. The innate immune system senses tissue damage and translates the information to other repair and defense systems in the body by stimulating wound repair and angiogenesis, as well as activating adaptive immunity [14]. Inflammasome signaling is followed by IL-1β processing and secretion in dendritic cells, macrophages, and epithelial cells [15,16,17,18,19,20]. Taken together, these events are associated with concomitant radiation-induced pneumonitis and fibrosis. In this study, we selected a clinical SBRT-treated animal model to better understand the therapeutic effects of bvPLA_2_ on radiation pneumonitis.

Our recent studies demonstrated that bee venom PLA_2_ possesses a strong capability to increase the population of regulatory T cells (Tregs) [21,22]. Tregs are potent immune modulatory cells and maintain tolerance toward autoantigens, and are also key immune suppressors for preventing overwhelming immune responses. Notably, bvPLA_2_ induced Tregs and caused immunosuppressive effects via Tregs in the lungs [23]. Based on these previous results, we hypothesized that bvPLA_2_ might be able to alleviate radiation-induced pneumonitis, mediated by Treg induction. In this study, we provide strong evidence that bvPLA_2_ has potential therapeutic use to reduce radiation-induced lung inflammation.

## 2. Results

### 2.1. The Effect of Bee Venom PLA_2_ on Histological Changes in Irradiated Lung Tissue

**Figure 1:**
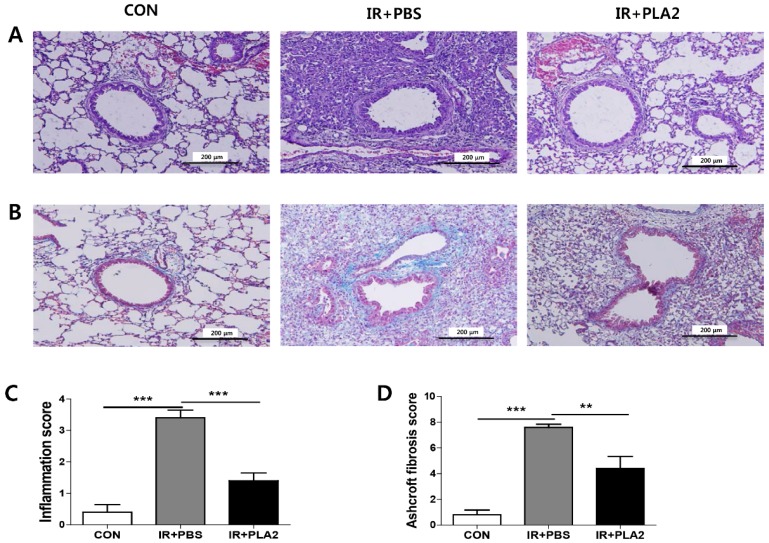
Effect of bee venom PLA_2_ on lung inflammation and lung fibrosis. (**A**) The lung sections were stained with H&E. Representative images of magnifications (×200) at day 21. (**B**) The lung sections were stained with Masson’s trichrome. Representative images of magnifications (×200) at day 21. (**C**) Quantification of inflammatory foci. (**D**) Quantification of fibrotic foci. Data are expressed as the mean ± SEM (** *p* < 0.01, *** *p* < 0.001 *versus* IR+PBS; *n* = 7–10). CON: non-irradiation, IR+PBS: irradiation + PBS, IR + PLA2: irradiation + bee venom PLA_2_.

To evaluate the effects of bvPLA_2_ on radiation-induced lung injury, a single dose of 75 Gy of X-rays was delivered to the left lung in a single fraction. Lung sections stained with H&E revealed that focal irradiation induced the formation of an intra-alveolar hyaline membrane and inflammatory cell infiltration (Figure 1). The alveolar inflammatory score of the IR+PBS group was significantly higher compared with the control (Figure 1C). By contrast, the IR+PLA_2_ group showed less tissue damage, such as the formation of an intra-alveolar hyaline membrane and inflammatory cell infiltration (Figure 1A). Masson’s trichrome staining revealed that the IR+PBS group showed significant increases in collagen deposition and number of fibrotic foci (Figure 1B,D). Treatment with bvPLA_2_ significantly reduced collagen deposition (Figure 1B,D).

### 2.2. The Effect of Bee Venom PLA_2_ on Pulmonary Inflammation

**Figure 2:**
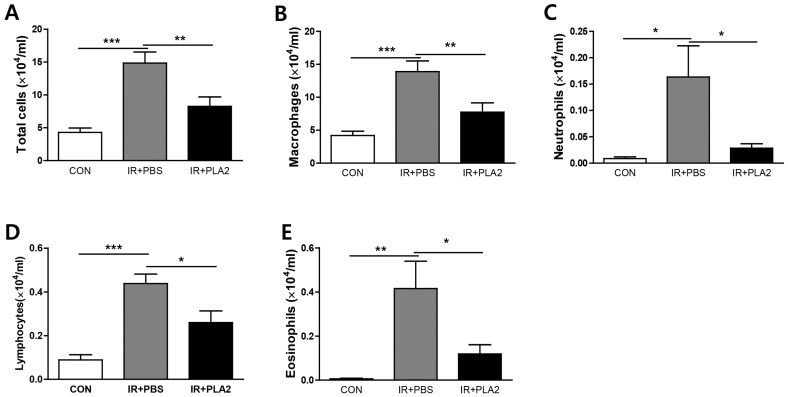
Effect of bee venom PLA_2_ on immune cell profiles in BAL fluid. The number of total cells (**A**); macrophages (**B**); neutrophils (**C**); lymphocytes (**D**) and eosinophils (**E**) were determined in BAL fluid. Data are expressed as the mean number of cells ± SEM (* *p* < 0.05, ** *p* < 0.01, *** *p* < 0.001 *versus* IR+PBS; *n* = 7–10). CON: non-irradiation, IR+PBS: irradiation + PBS, IR+PLA2: irradiation + bee venom PLA_2_.

To elucidate whether bvPLA_2_ influences immune cell infiltration into the lung in radiation-induced inflammation, BAL fluid mice were evaluated three weeks after treatment with 75 Gy of radiation. A significant increase in the influx of total cells, macrophages, neutrophils, lymphocytes, and eosinophils was observed in the IR+PBS group compared to the control group. The bvPLA_2_ treatment group exhibited significantly decreased numbers of immune cells, including macrophages, neutrophils, lymphocytes, and eosinophils in BAL fluid, compared with the IR+PBS group (Figure 2).

### 2.3. The Effects of Bee Venom PLA_2_ on Inflammation- and Fibrosis-Related Gene Expressions in Irradiated Lung Tissue

**Figure 3:**
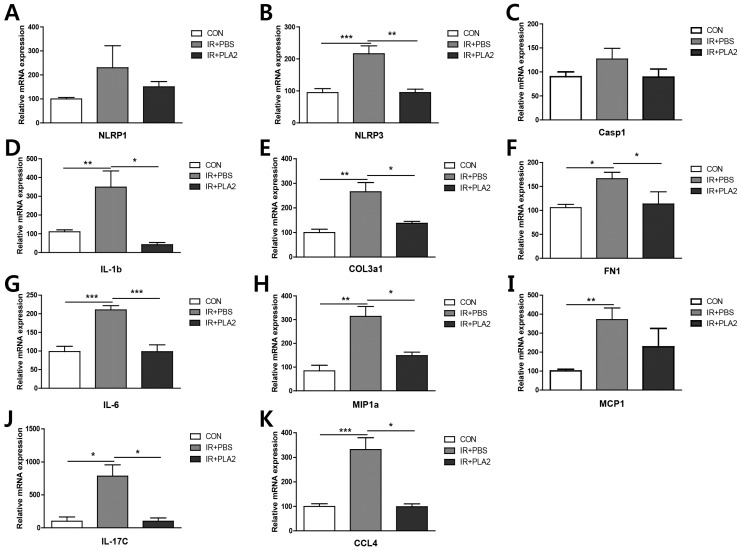
Effect of bee venom PLA_2_ on mRNA levels in lung tissue. (**A**) *Nlrp3*; (**B**) *Nlrp1*; (**C**) *Casp1*; (**D**) *Il-1b*; (**E**) *Col3a1*; (**F**) *Fn1*; (**G**) *Il-6*; (**H**) *Mip1a*; (**I**) *Mcp1*; (**J**) *Il-17c*; (**K**) *CCL4*. Data are expressed as the mean ± SEM (* *p* < 0.05, ** *p* < 0.01, *** *p* < 0.001 *versus* IR+PBS; *n* = 7–10). CON: non-irradiation, IR + PBS: irradiation + PBS, IR + PLA2: irradiation + bee venom PLA_2_.

**Figure 4:**
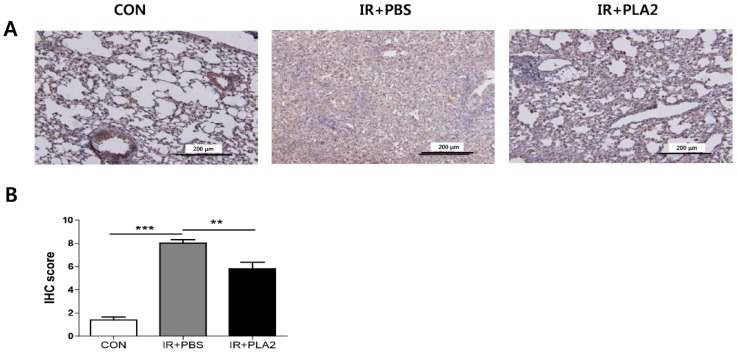
Effect of bee venom PLA_2_ on TGF-β1 expression in lung tissue. Lung sections were stained with TGF-β1 using immunohistochemistry at day 21 after irradiation. CON: non-irradiation, IR+PBS: irradiation + PBS, IR + PLA2: irradiation + bee venom PLA_2_. (**A**) Representative images of magnifications (×200). (**B**) Quantification of TGF-β1 expression foci, ×200. Randomly selected fields of view for each slide were scored for area and intensity of positively stained (brown) cytoplasm and cell membrane. The intensity scores for positively stained areas were divided into 4 groups: (1) no appreciable staining = 0; (2) barely detectable staining = 1; (3) readily appreciable brown staining = 2; and (4) dark brown staining = 3. The total score was calculated by adding the intensity scores from three independent slides in each sample, resulting in a final score of 0 to 9. For statistical analysis, scores of 3 to 9 were defined as positive expression, while scores of 0 to 2 were defined as negative expression. Data are expressed as the mean ± SEM (** *p* < 0.01, *** *p* < 0.001 *versus* IR+PBS; *n* = 7–10).

Gene expression profiles in the lung were assessed by real-time PCR. The expression of inflammasome- (*Nlrp1*, *Nlrp3*, *Il-1b*, and *Casp1*), chemokine- (*Mip1a, Mcp1*, and *CCL4*), cytokine- (*Il-6* and *Il-17c*), and fibrosis-related (*Col3a1* and *Fn1*) genes were increased in the IR+PBS group (Figure 3). However, treatment with bvPLA_2_ significantly reduced inflammasome-, chemokine-, cytokine-, or fibrosis-related genes in lung tissue, compared to the IR + PBS group (Figure 3). We also performed IHC staining for TGF-β1 (Figure 4), because TGF-β plays an important role in the development of radiation pneumonitis and radiation fibrosis [24]. The histological results also demonstrated that the TGF-β1 positive areas of the X-ray irradiated lung were significantly decreased by bvPLA_2_ treatment (Figure 4B).

### 2.4. The Role of Tregs in the Therapeutic Effects of Bee Venom PLA_2_ after Radiation-Induced Lung Inflammation

**Figure 5:**
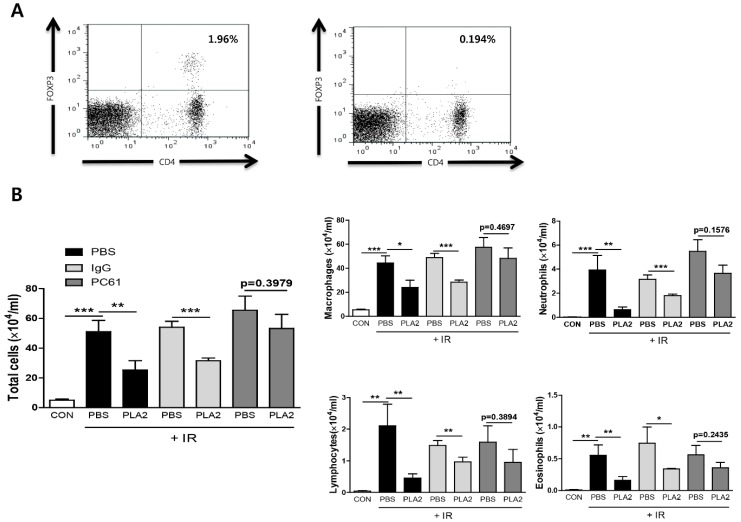
Effect of bee venom PLA_2_ and CD4^+^CD25^+^ Treg deficiency on immune cell profiles in BAL fluid. (**A**) Confirmation of CD4^+^CD25^+^ Treg depletion (left: rat IgG treated, right: PC61 treated). (**B**) The number of immune cells was counted in BAL fluid. CON: non-treated, PBS: irradiation + PBS, PLA2: irradiation + bee venom PLA_2_, IgG: rat IgG, PC61: anti-CD25 antibody. Data are expressed as the mean number of cells ± SEM (* *p* < 0.05, ** *p* < 0.01, *** *p* < 0.001 *versus* indicated group; *n* = 5).

A previous report demonstrated that Treg depletion by an anti-CD25 antibody injection abolished the anti-inflammatory effects of bee venom in asthma, multiple sclerosis, and lupus nephritis [25,26,27]. Furthermore, it has been confirmed that bvPLA_2_ induced Tregs from naïve CD4 positive T cells [21] and that Tregs are critical to lung fibrosis [28,29]. To determine whether Tregs were involved in the effects of bvPLA_2_ on radiation-induced pneumonitis, mice were injected with the PC61 antibody to deplete Treg *in vivo*. The efficacy of Treg depletion (≥90%) was confirmed prior to irradiation (Figure 5A). The Treg-depleted model system showed a distinct role for Tregs in the effect of bvPLA_2_ on immune cell infiltration in lung tissue. BvPLA_2_ treatment showed an obvious effect on the influx of total cells, macrophages, neutrophils, lymphocytes, and eosinophils into BAL fluid in control IgG antibody injected mice. In anti-CD25 antibody-injected mice, however, bvPLA_2_ showed a tendency to decrease the number of such infiltrated cells, but this was not statistically significant (Figure 5B). These results suggest that Tregs, at least in part, contribute to the therapeutic effects of bvPLA_2_ in radiation-induced lung inflammation.

## 3. Discussion

Radiation-induced lung injury is characterized by airway inflammation and occurs in up to 30% of patients who receive irradiation for lung tumor treatment and in approximately 15% of patients with other thoracic cancer, which is now a leading and increasing cause of high morbidity and mortality worldwide [30,31,32]. The goal of this study was to elucidate the effects of bvPLA_2_ on IR-induced lung injury. The results of the present study demonstrate that exposure to radiation induces structural changes in terms of inflammation in the lung. In addition, we observed collagen deposition in IR+PBS mice, suggesting an overlap between pneumonitis and fibrosis [33]. The lung tissue of the IR+PBS group showed classical appearances of acute lung injury. By contrast, the bvPLA_2_-treated group showed less inflammatory cell infiltration in the alveolar space and bronchial lumen.

Bee venom has long been used in alternative medicine to treat a variety of immune-related diseases, such as multiple sclerosis and rheumatoid arthritis, and possesses strong immune modulatory effects [1,2,3,4,5,6,34,35]. Our recent studies have demonstrated that bvPLA_2_ possesses a strong capability to increase the population of Tregs and to decrease the inflammatory responses [21,22]. PLA_2_ is a lipolytic enzyme that produces lysophospholipid and free fatty acids, mainly arachidonic acid, which is a precursor of different bioactive lipid mediators [36]. PLA_2_ is present in the venoms of snakes, bees, mammals, and scorpions. It has been classified into 15 distinct groups and 4 main types, according to its structure, source, and function [36]. PLA_2_ derived from bee venom belongs to group III secretory PLA_2_ [8]. Each PLA_2_ has an intrinsic function separate to the common function of these enzymes, which causes cleavage of the sn-2 acyl bond of phospholipids. Von Allmen *et al.* demonstrated that PLA_2_-IID secreted from mouse Tregs promoted the differentiation of Tregs, and inhibited disease development of colitis and multiple sclerosis [37]. For the proper function of bvPLA_2_, catalytic activity was not required, yet, in our previous study, heat-inactivated bvPLA_2_ lost its effect for Treg differentiation [21,22]. In the current study, we used catalytically active bvPLA_2_ and verified its therapeutic effects on radiation-induced lung inflammation; however, we did not compare the effects between enzymatically active- and inactive-forms of bvPLA_2_. A comparison of the effects of wild-type and H34Q [38] point mutated-recombinant bvPLA_2_ would confirm whether the enzymatic activity is involved, or not, in the observed anti-inflammatory action. Recently, Palm *et al.* demonstrated that bvPLA_2_ induced a Th2 response, which was dependent on MyD88 [39]. The necessity of the catalytic activity of bvPLA_2_ for its therapeutic use remains unclear. Even though we focused on the Treg-related mechanism in this study, the anti-inflammatory effect of bvPLA_2_ on radiation-induced inflammation might be partly due to anti-inflammatory cytokines, such as IL-4 [40]. In some part, different Th response could be dependent upon the used dosage of bvPLA_2_ for each experiment, since the binding affinity of bvPLA_2_ for receptors, such as MyD88 and CD206, is different. In practice, we used a much lower dosage of bvPLA_2_ (1/12 to 1/25 of the dosage used by Palm *et al.*) [39]. The binding capacity to CD206 and the enzymatic activity of each venom’s PLA_2_ may play a role in PGE2 production within dendritic cells. Baratelli *et al.* showed that PGE2 induces Foxp3 gene expression and Treg function in human CD4^+^ T cells [41]. To elucidate the detailed function of bvPLA_2_, further mechanistic studies are required.

The anti-inflammatory actions of Tregs can protect against lung injury by inhibiting the influx of macrophages, neutrophils, lymphocytes, and eosinophils, which act as a source of pro-inflammatory cytokine production [42,43]. Tregs can inhibit fibrosis, and, moreover, impairment of Treg is shown in fibrosis in the lungs and other organs [28,29,44]. In our previous study, we demonstrated that bvPLA_2_ binds to the CD206 receptor on dendritic cells, and this binding induced COX2 expression and resulted in PGE2 secretion [21]. PGE2 directly induced Treg differentiation by binding to the EP2 receptor on CD4^+^ T cells. Ultimately, the Treg population was increased through this pathway. In addition, it is suggested that PGE2 increases cAMP in fibroblasts through EP2 receptors, which inhibited fibroblast proliferation, stimulated the death of fibroblasts, and inhibited extracellular matrix protein synthesis, resulting in protection against bleomycin-induced pulmonary fibrosis [45,46]. We have also shown that bvPLA_2_ induced Treg and showed immunosuppressive effects via Treg in the lung of OVA-induced asthmatic mice [23]. Our findings show that, in line with our previous data, the therapeutic effect of bvPLA_2_ is associated with Treg in a radiation-induced lung inflammatory model. However, the number of infiltrated inflammatory cells into the lung tissue was decreased by bvPLA_2_ treatment in a Treg deficient system, though it was not statistically significant. The result might come from incomplete Treg deletion, or it implies that there is another cellular mechanism beyond Treg for the anti-inflammatory effects of bvPLA_2_. For example, CD206^+^ macrophages may participate in the effects of bvPLA_2_ in radiation-induced lung inflammation. The mechanism of bvPLA_2_ involved in radiation-induced lung injury remains, for the most part, unknown.

The observed inflammatory changes were accompanied by an increase in cytokines, such as IL-1β and IL-6, as is observed in pneumonitis, which are associated with impaired lung function [47,48,49,50,51,52,53]. In addition, profibrotic cytokines, such as TGF-β and IL-13, contribute to pulmonary fibrosis by promoting the conversion of fibroblasts to myofibroblasts [24,54,55,56]. Specifically, TGF-β plays an important role in the development of radiation pneumonitis and radiation fibrosis [24]. These cytokines also activate macrophages [55,56]. In the present study, the bvPLA_2_-treated group showed a suppression of these cytokines and macrophages in lung tissue. Our recent study demonstrated that radiation can injure the cells that activate the *NLRP3* inflammasome [33]. The failure to clear the byproducts of cellular damage induced by radiation leads to a hyperactivation of the innate immune system and fibrosis in the lungs [57]. CD4^+^ T cells abolish macrophage NLRP1 and *NLRP3* inflammasome-mediated caspase-1 activation and subsequent interleukin 1β release [58]. CD4^+^CD25^+^ Tregs are a subset of the thymus-derived CD4^+^ T cell population that play a crucial role in immune regulation by inhibiting the production of cytokines via their constitutive expression of the transcription factor Foxp3 [59,60]. Consistent with these reports, our results show that the *NLRP3* inflammasome-related (*Nlrp1*, *Nlrp3*, *Il-1b*, *and Casp1*) and fibrosis-related (*Col3al and Fn1*) genes are reduced. These findings suggest that the protective effects of bvPLA_2_ are associated with the suppressed hyperactivation of the innate immune system.

Taken together, the results of this study suggest that bvPLA_2_ is an effective target for the regulation of Tregs to significantly ameliorate radiation-induced lung inflammation and fibrosis. BvPLA_2_ is, as such, a potential candidate for treating radiation-induced inflammation and fibrosis.

## 4. Materials and Methods

### 4.1. Irradiation Equipment

Radiation was delivered to a small volume of the left lung, using an image-guided small-animal irradiator, an X-RAD 320 (Precision, North Branford, CT, USA). This irradiator is equipped with a collimator system that is composed of 3.5-cm-thick copper to produce focal radiation beams, an imaging subsystem consisting of a fluorescent screen coupled with a charge-coupled-device camera, and a manually operated stage. The collimators generate a cone beam that ranges from 1 to 7 mm in diameter. The percentage depth doses were measured with GAFCHROMIC EBT2 film [61]. To mimic clinical stereotactic ablative radiotherapy conditions by irradiating only a small tissue volume, we selected 3-mm collimators.

### 4.2. Mouse Irradiation

All the experiments and animal handling procedures in this study were approved by the Animal Experimental Ethics Committee of the Kyung Hee University and Yonsei University (permit number: 2014-0164-1, approved on 06/26/2014) and performed according to local guidelines. C57BL/6 female mice (6 weeks old, weighing 20–25 g) were purchased from Charles River Korea (Orient Bio, Seongnam, South Korea). The mice were housed (*n* = 5 in each cage) and allowed to acclimatize for 1 week prior to treatment. A single dose of 75 Gy was delivered to the left lung in a single fraction. In our previous study, we found that a radiation dose of 75 Gy induced inflammation after 3 weeks. The mice were randomly divided into 3 groups (*n* = 7–10/group): (1) control group: Negative control mice received an intraperitoneal (i.p.) injection of phosphate-buffered saline (PBS) on day 7, 10, 12, 14, 17, and 19; (2) IR+PBS group: Mice were exposed to a single dose of 75 Gy delivered to the left lung in a single fraction and then i.p. injection with PBS on day 7, 10, 12, 14, 17, and 19; (3) bvPLA_2_ group: Bee venom-derived PLA_2_ (Sigma-Aldrich, St. Louis, MO, USA) was dissolved in PBS (1 mg/mL) for the stock solution. Stocks were stored −20 °C and diluted with PBS before administration. The mice were administered with bvPLA_2_ (0.2 mg/kg body weight.) via i.p. injection on day 7, 10, 12, 14, 17, and 19 after IR. During irradiation, the mice were anesthetized with an i.p. administered mixture of 30 mg/kg zoletil and 10 mg/kg rompun. At day 21, mice were sacrificed by CO_2_ asphyxiation, and lung tissues were collected for analyses. No mice died during the experiment.

### 4.3. Depletion of CD4^+^CD25^+^ Regulatory T Cells In Vivo

Anti-mouse CD25 rat IgG1 (anti-CD25; Clone PC61) was generated in-house from hybridomas obtained from American Type Culture Collection (Manassas, VA, USA). Before irradiation, we confirmed the efficacy of CD4^+^CD25^+^ Treg depletion by flow cytometry. We used FOXP3^EGFP^ mice, with PE-anti-mouse CD25 and APC-mouse CD4 antibodies. A single dose of 75 Gy was delivered to the left lung in a single fraction. The C57BL/6 female mice were randomly divided into 7 groups (*n* = 5/group): (1) control group; (2) IR + PBS group; (3) IR + PBS + total rat IgG group; (4) IR + PBS + PC61 group; (5) IR + bvPLA_2_ group; (6) IR + bvPLA_2_ group+total rat IgG group; and (7) IR + bvPLA_2_ group+PC61 group. A dose of 0.25 mg of the anti-CD25 antibody and total rat IgG was injected on days 0 and 7; (1) control group: Negative control mice received an i.p. injection with PBS alone on days 1, 4, 6, 8, 11, and 13; (2) IR + PBS groups: Mice were exposed to a single dose of 75 Gy delivered to the left lung in a single fraction and then i.p. injection with PBS alone on days 1, 4, 6, 8, 11 and 13; (3) bvPLA_2_ groups: Mice were administered with bvPLA_2_ (0.2 mg/kg body weight.) via i.p. injection on days 1, 4, 6, 8, 11 and 13 after IR. During irradiation, the mice were anesthetized with an i.p. administered mixture of 30 mg/kg zoletil and 10 mg/kg rompun. At day 14, the mice were euthanized using CO_2_ asphyxiation, and lung tissues were collected for analysis.

### 4.4. Analysis of Lung Inflammatory Cells

PBS was gradually infused into the lungs and then withdrawn via a cannula that had been inserted into the trachea. This procedure was repeated 3 times, and the lavages were collected. Following this, collected BAL fluid was centrifuged at 1300 rpm for 10 min. The supernatants were stored at −80 °C until further analysis, and the cell pellets were resuspended in 1 mL PBS and adhered to glass slides using cytocentrifugation. The numbers of total and differential cells in the BAL fluid were then determined in a hemocytometer, using trypan blue exclusion. Cells in BAL fluid were distinguished as macrophages, neutrophils, lymphocytes, and eosinophils by Diff-Quick staining [62]. In brief, we classify immune cells by size, shape, and color by staining. Eosinophils were stained using Eosin. Macrophages, neutrophils and lymphocytes were stained using hematoxylin. Cells with single, large, round, or indented nuclei were classified as macrophages. Cells with a polymorphonuclear nucleus, with several lobes were sorted as neutrophils. Cells with a clear perinuclear zone around the nucleus were categorized as lymphocytes.

### 4.5. Preparation of Lung Tissue for Histology and Immunohistochemistry

Lung tissues were fixed in a 4% paraformaldehyde solution and then embedded in paraffin. For histological examination, 4-μm sections of lung tissue were stained sequentially with hematoxylin and eosin (H&E), Masson’s trichrome (MT), and immunohistochemical (IHC) stains. For TGF-β1 detection, lung tissues were treated with 0.3% H_2_O-methanol for 20 min to block endogenous peroxidase. Subsequently, the sectioned tissues were incubated at 4 °C overnight with an anti-TGF-β1 primary antibody (1:100 dilution; ab64715, Abcam). After the slides were incubated with biotinylated secondary antibody and then with a VECTASTAIN^®^ ABC Reagent (Vector Laboratories, Burlingame, CA, USA), the color was developed with 3,3'-diaminobenzidine tetrachloride (DAB; Zymed Laboratories, San Francisco, CA, USA). After IHC staining, the slides were counterstained with Harris’s hematoxylin for 1 min and then mounted with Canada balsam (Show Chemical Co. Ltd., Tokyo, Japan).

### 4.6. Histopathology and Immunohistochemistry Scoring

For histopathological evaluation, sections were stained with H&E and MT stains, and scored for the number of inflammatory or fibrotic foci, respectively. For IHC evaluation, sections were stained with TGF-β1 staining. Slides were assessed according to a dual rate semi-quantitative method carried out by 5 independent pathologists who were blinded to the sample data [63]. Randomly selected fields of view for each slide were scored for area and intensity of positively stained (brown) cytoplasm and cell membrane. The intensity scores for positively stained areas were divided into 4 groups: (1) no appreciable staining = 0; (2) barely detectable staining = 1; (3) readily appreciable brown staining = 2; and (4) dark brown staining = 3. The total score was calculated by adding the intensity scores from three independent slides in each sample, resulting in a final score of 0 to 9. For statistical analysis, scores of 3 to 9 were defined as positive expression, while scores of 0 to 2 were defined as negative expression.

### 4.7. Real-Time Quantitative PCR Analysis

RNA was isolated from lung tissue using an RNeasy Mini Kit (Qiagen, CA, USA) according to the manufacturer’s instructions, and the RNA was quantified using a NanoDrop (ND-1000; NanoDrop Technologies, Inc., Wilmington, DE, USA). Real-time quantitative PCR was performed using LightCycler 480 SYBR Green I Master mix and a Light Cycler 480 real-time PCR machine (Roche Applied Science, Indianapolis, IN, USA). The expression levels of transcripts were evaluated using Light Cycler 480 software. The transcript levels of β-actin were used for sample normalization. Data were obtained from 3 independent experiments and are represented as average ± standard error. The sequences of the mouse primers were as follows: *Nlrp1* (FW 5'-catggctggttcctaccact-3'; RW 5'-ccaaccaccatgtgactctg-3'), *Nlrp3* (FW 5'-atgctgcttcgacatctcct-3'; RW 5'-gtttctggaggttgcagagc-3'), *Casp1* (FW 5'-cacagctctggagatggtga-3'; RW 5'-ggtcccacatattccctcct-3'), *Il-1b* (FW 5'-gcccatcctctgtgactcat-3'; RW 5'-aggccacaggtattttgtcg-3'), *Ccl4* (FW 5'-cccacttcctgctgtttctc-3'; RW 5'-gtctgcctcttttggtcagg-3'), *Col3a1* (FW 5'-accaaaaggtgatgctggac-3'; RW 5'-gacctcgtgctccagttagc-3'), *Fn1* (FW 5'-acagagctcaacctccctga-3'; RW 5'-tgtgctctcctggttctcct-3'), *Il-6* (FW 5'-ccggagaggagacttcacag-3'; RW 5'-tccacgatttcccagagaac-3'), *Mip1a* (FW 5'-atgaaggtctccaccactgc-3'; RW 5'-gatgaattggcgtggaatct-3'), *Mcp1* (FW 5'-ccaatgagtaggctggaga-3'; RW 5'-tctggacccattccttcttg-3'), *Il-17c* (FW 5'-aggtgctggaagctgacact-3'; RW 5'-catccacgacacaagcattc-3'), *Foxp3* (FW 5'-tcttgccaagctggaagact-3'; RW 5'-ggggttcaaggaagaagagg-3'), and β*-actin* (FW 5'-gatctggcaccacaccttct-3'; RW 5'-ggggtgttgaaggtctcaaa-3').

### 4.8. Statistical Analysis

Statistical analysis of the data was conducted using Prism 5 software (GraphPad Software Inc., San Diego, CA, USA). All of the values are presented as the mean ± SEM (standard error of the mean). The statistical significance was assessed by one-way ANOVA followed with Turkey’s test with randomly selected samples. A *p*-value < 0.05 was considered statistically significant.
